# The Digitized Ecosystem of Tourism in Europe: Current Trends and Implications

**DOI:** 10.1007/978-3-030-65785-7_34

**Published:** 2020-11-28

**Authors:** Norman Schaffer, Martin Engert, Guido Sommer, Jasmin Shokoui, Helmut Krcmar

**Affiliations:** 1 Department for Informatics, Technical University of Munich, Garching bei München, Bayern Germany; 2 Smart Tourism Education Platform (STEP) College of Hotel and Tourism Management, Kyung Hee University, Seoul, Korea (Republic of); 3 Department of Tourism and Service Management, MODUL University Vienna, Vienna, Wien Austria; 4 Fortiss GmbH, Munich, Germany; 5 Faculty for Informatics, Technical University Munich, Garching, Germany; 6 Faculty for Tourism Management, University of Applied Sciences Kempten, Kempten, Germany

**Keywords:** Tourism ecosystem, Digital transformation, Value network

## Abstract

Emerging digital technologies enable the creation of new services and business models, leading to ecosystems’ continuous change. In the tourism industry, new digital-savvy players like Airbnb have entered and created entirely new market segments, while many existing players are challenged to redefine their business logic. However, the literature does not provide a generic overview of the value network in tourism, including new market players, and their ways of interacting. Therefore, this paper develops a current overview of the value network of the European tourism ecosystem. By analyzing the business models and value streams of 704 European enterprises based on Crunchbase data, we identified 27 distinct roles and their respective interrelations in the domain. To validate the proposed value network, we conducted five expert interviews. Our results highlight the continuously growing importance of intermediaries in tourism. Furthermore, new technology players offer new opportunities for innovative services, creating high dynamism in the industry. Nonetheless, local entities, such as residents and communities, play a central role in European tourism and need to be included in experiences provided to tourists. Scholars and practitioners might use the results to identify disruptive actors and opportunities for innovation and niche creation. Additionally, the results can be used as a basis for further analysis of the ecosystem’s ongoing changes induced through technological advancements or external events such as the COVID-19 pandemic.

## Introduction

Digital technologies transform the daily lives of consumers, companies, and the structure of entire ecosystems [32]. Through digital innovation and resulting digital transformation, new kinds of interactions and value creation emerge [40]. These changes challenge organizations in all industries, from product-centric to service-centric [39].

No exception, the tourism industry has undergone fundamental changes in the last two decades. Touristic behaviors and experiences have changed, and powerful actors have ramped up their operations globally. Players like Airbnb encourage private entities to lease their accommodations to fellow travelers, while Expedia and other aggregators offer one-stop-shops for services ranging from transportation to accommodation, rental cars, and on-site experiences. Tripadvisor is one of the largest online communities for exchanging and rating all kinds of touristic players, destinations, and activities. Due to current megatrends such as the restructuring of economies, the role of data, and shifts of global power, the tourism industry is likely to continue to face disruptive changes [38].

Consumers, businesses, and various other public and public-private players interact with information and communication technologies in new ways, leading to dynamic interactions among different touristic stakeholders [11]. These interactions among multiple actors within the ecosystem lead to the co-creation of value [16, 17]. However, tourism ecosystems are particularly dynamic, with new local and global players emerging during the past few decades [11].

To thrive in the ecosystem and drive innovation, however, understanding the ecosystem’s active position and purpose (subsumed as role), and interrelations between them, is key. Furthermore, the knowledge of actors and value streams enables the development of more sustainable business approaches as the value is co-created among multiple stakeholders [33]. Practitioners need to understand the ecosystem’s active roles to identify disruptive actors or potential business opportunities. Current research often focuses on specific technologies, such as augmented reality (AR) or specific players like hotels, but does not consider the ecosystem as a whole [11]. Furthermore, the high dynamic of the ecosystem, its actors, and their interactions make understanding the status quo difficult. Indeed, detailed identification of ecosystem actors and their interactions is required [11], and [13] even call for transformative work on the impact of digital technologies on tourism beyond COVID-19.

However, the research is still missing a holistic analysis of the current and ongoing transformation of the tourism ecosystem.

Therefore, this paper aims to answer the following research question: *What are the generic roles and their interactions in the European tourism ecosystem?*

To answer this question, we followed the research approach of [30, 31] and [32] to identify 27 generic roles derived from analyzing 704 companies from the database Crunchbase. To visualize the ecosystem and understand the interrelations between involved actors, we created an e3-value model. The e3-value method is a business modeling method to elicit, analyze, and evaluate business ideas from an ecosystem perspective [1, 10]. Based on this ecosystem, we discuss the strategic implications of today’s tourism ecosystem, such as aggregation of intermediaries and the growing importance of data-based services.

## Related Work

Digital transformation led to the emergence of highly complex relationships among actors and industry players who can no longer be grouped into suppliers, customers, and competitors [7, 24]. Having evolved from this classical value chain perspective, the concept of ecosystems goes beyond linear relationships and describes a group of loosely interacting and interdependent actors [14]. Firms such as Google, Amazon.com, and Alibaba leverage a large network of actors that provide complementary products and services. [35] defines “business ecosystems” as “[…] the community of organizations, institutions, and individuals that impact the enterprise and the enterprise’s customers and supplies.” Digitally-enabled transformation of many different industries, from linear value chains to highly interrelated and interdependent ecosystems, also affects the tourism ecosystem. Transferring this notion of “ecosystems” to tourism is justified due to the involved actors’ complexity and interrelatedness, as [2] showed regarding tourism destinations. In this study’s context, extending this view when “zooming-out,” the generic term *tourism ecosystem* describes not only destinations and their actors but also the respective tourism ecosystem and its actors in any one country and region. Therefore, the tourism ecosystem in Europe as this study’s unit of analysis entails all stakeholders and their interactions and also the resulting interrelations in this particular region. Prior work has introduced different concepts to describe ongoing changes and consequences induced by digital technologies in tourism, and the next sections present these concepts in more detail.

Accounting for services’ increasing importance and the resulting servitization, researchers have introduced the term *tourism service ecosystem.* It follows the notion of Service-Dominant Logic (SD-Logic) (cf. [36] and [37]) to describe actors participating in resource integration and service exchange. These activities are enabled and constrained by endogenously generated institutions and institutional arrangements, and interlocking service ecosystems where value is co-created in the context of tourism [5]. This perspective allows researchers to account for increasing levels of value co-creation among actors within tourism ecosystems. In particular, it acknowledges digital technologies’ role of enabling increased levels of servitization and value co-creation.

Furthermore, prior work on the use and impact of information and communication technologies in tourism led to the concept of *e-tourism*, used to describe digital technologies’ wider impacts on tourism in general, thereby taking the perspective of tourists and businesses alike [8]. In the context of e-tourism, concepts such as business models were introduced to understand individual firms’ business logic [28, 29]. Furthermore, research on e-tourism highlights the role of ecosystems and their dynamics [3, 26].

Based on the early term *e-tourism*, the literature accounting for digital technologies’ ubiquity and the resulting connectedness in tourism referred to this advanced phenomenon as *smart tourism* [12, 15]. Smart tourism offers an integrated perspective on data, technological infrastructure, and the creation of business value. Moreover, *smart tourism ecosystems* (STEs) describe tourism systems that take advantage of smart technology in several ways. STEs are characterized by intensive information sharing and value co-creation among actors and include a variety of “species” such as touristic and residential consumers, tourism suppliers, tourism intermediaries, and others [11] who co-create value within tourism ecosystems. An example of a local smart tourism ecosystem is the region of Salerno, Italy. Political administrative structures, such as the municipality and provincial tourism authority, periodically contact hosts, update them on events in the city, and invite them to regular meetings [27].

While the terms above describe different phenomena resulting from the impact of digital technologies on tourism in general, prior work was reluctant to analyze and discuss implications for a tourism ecosystem as the unit of analysis. Thus, this work aims to uncover advanced digital technologies’ effects on different touristic and increasingly technology-focused actors and their respective interrelations subsumed into the tourism ecosystem in Europe.

## Research Approach

We followed the research design proposed by [30, 31], and [32]. We first identified active actors’ roles in the tourism industry and the value streams between them. Second, we converted the roles and interrelations into an e3-value-network to visualize the derived ecosystem. Third, we validated the model with semi-structured interviews of experts from the tourism domain and adapted the ecosystem according to their feedback.

First, we used the database Crunchbase,^1^ which provides extensive information for existing companies and startups [19]. We extracted a dataset on September 13, 2018, applying the category “tourism” and the location “headquarter in Europe.” This provided 851 European companies categorized as tourism companies according to Crunchbase. We excluded 124 companies no longer in existence. Then, we excluded further 23 companies classified into several categories close to tourism, but their value proposition was neither directly nor indirectly relevant to the industry. Thus, their relation to the tourism domain was nonexistent according to our understanding of the respective companies. Our final sample consisted of 704 companies. Using this sample, we performed a structured content analysis to develop categories inductively, i.e., generic roles, based on [20] and [21]. In addition to the information available in Crunchbase, we used publicly available information from the Internet, such as homepages of respective companies as well as reports, press articles, and annual reports. To ensure coding consistency, three experts individually coded the 704 organizations. To become familiar with the coding scheme, in the beginning, each expert coded 50 companies individually. Afterward, we compared and discussed these 50 organizations’ coding for calibration purposes. Once this step was completed, all experts coded half the organizations independently, followed by another calibration step. Based on this, the remaining organizations were again coded independently by the three experts. All authors discussed the coding and discrepancies, eventually eliminating individual disparities and reaching consensus [9]. Then, we derived interrelations and value streams between generated roles through structured content analysis [20]. The concept of value systems was introduced by Porter and further extended to value networks to analyze industries, its’ roles, inherent functions, activities, and their interrelations: value streams [6, 24]. To derive the value streams, we again used all available information. Primarily, we checked explicit interrelations these companies have with other companies, using the information provided about partners, customers, and suppliers of individual organizations on their public homepages and in other sources, such as newspapers.

In the second step, we visualized the ecosystem by developing an e3-value network based on coded generic roles and their interrelations. The method is suitable for abstracting roles from similar organizations and for modeling large ecosystems with a variety of stakeholders [32]. Our result was a generic e3-value network, i.e., the tourism ecosystem of Europe.

Third, from June to July 2020, we evaluated the developed ecosystem by conducting five interviews with representatives of the tourism industry. Among the experts were a managing director of a large online portal for holiday apartments, a former executive of the German National Tourism Board, a former executive of a large tour operator, a hospitality expert, and the head of R&D of a large tour and activity portal. Due to the experts’ extensive experience and their different roles and approaches to the industry, a variety of interrelations were revised, and nine roles were added or changed in the final ecosystem. In an additional step to validate our findings, we randomly extracted 50 American-based tourism organizations from Crunchbase in October 2020. We chose American based companies due to the much easier accessibility and comprehensibility of additional information online via websites and blogs. The research team extended this sample, to deliberately include the well-known players Airbnb, Booking.com, and Tripadvisor. Two researchers then successfully coded all 53 companies into existing roles. As such, the identified roles seem suitable for non-European players, increasing the validity and generalizability of our findings outside the European tourism ecosystem.

## Results

This section presents our research results. First, we introduce derived roles active in the ecosystem. Second, we present an overview of the tourism value network. After that, we discuss current trends and innovation patterns in the ecosystem.

### The Generic Tourism Ecosystem

Picturing the tourism ecosystem requires understanding which individuals belong to it. Based on our structured content analysis of 704 organizations’ Crunchbase data, Table 1 displays 27 roles identified in the ecosystem. Besides existing roles, we identified new roles that have emerged in recent years because of technological developments. Of course, one organization can act in different roles by offering different services to other players.

**Table 1.** Generic roles of actors in the tourism ecosystem

Role	Description	Example
Analytics Technology Providers	Companies providing analytics services or data insights. Their main value is collecting and processing touristic information that can be commercialized to other stakeholders inside the industry, or they sell insights from the data or analytics services that the customer can use	Milanamos Kido Dynamics Yourmyguide
Data Technology Providers	Providers of data and data sources, e.g., weather data, traffic data, capacities of regions, POIs, etc. Mostly, APIs are provided, which can be integrated into existing services	Amadeus Travel APIs GIATA
Digital Infrastructure Technology Providers	Providers of digital infrastructure, mostly platforms, to support service provision, e.g., booking-systems (mostly back-end solutions without direct contact with the tourist)	Amadeus IT Group
Disruptive Technology Providers: AR, VR, MR	Providers of disruptive technologies, offering AR, Virtual Reality (VR), or Mixed Reality (MR) services	Urban Time Travel
Disruptive Technology Providers: Artificial Intelligence	Providers of disruptive technologies, selling either services or software solutions based on Artificial Intelligence (mostly Machine Learning)	Surebot
Disruptive Technology Providers: Blockchain	Providers of disruptive technologies, selling either services or software solutions based on blockchain	Windingtree
Disruptive Technology Providers: Internet of Things	Providers of disruptive technologies, selling IoT-Solutions (cooperative work of physical and virtual objects)	Valpas Enterprises OY Yuubo
DMO (public & private)	Destination Marketing Organizations promote specific touristic regions to potential customers. Often, these are public or public-private partnerships	Allgäu GmbH visitBerlin
Intermediaries: Experiences	Leisure agents sell experiences, e.g., tours or museum visits to tourists by acquiring quotas from actual service providers. The physical service is performed by tourism experience providers	Tours&Tickets; Ceetiz
Intermediaries: Hospitality	Leisure agents sell Leisure services, e.g., hotel, camping, etc., to tourists by acquiring quotas from actual service providers. The physical service is performed by tourism experience providers	Hostelworld
Intermediaries: Tour Operators & Travel agents	Commercial providers of aggregated experiences, e.g., packaged tours. The physical service is performed by a tourism experience provider, and travel agents aggregate experiences into bundles	Trip Mule Classic Travel
Intermediaries: Transportation	Transportation agents sell transportation services, e.g., flights, to tourists by acquiring quotas from actual service providers. The physical service is performed by tourism experience providers	Urbo Solutions
Marketing and PR agencies	Specialized agencies for marketing and PR services in tourism promote concrete POIs, attractions, accommodations, or whole regions	Mylike
Online Communities: Content providers	Online communities providing touristic content	Vivere.travel Excursiopedia Live2Leave
Online Communities: Ratings	Online communities providing ratings of touristic destinations, activities, POIs, etc.	Mangrove TripAdvisor
Search Engine Optimization (SEO)	Companies offering services to navigate the Web, gathering travel options according to user requisites. SEOs redirect the user to vendor websites (no transactional process)	Flykt
Shared Accommodation	Accommodation provided by private entities, mostly locals, e.g., rental of a private apartment. In general, commercial platforms connect private and commercial entities	Farmbnb HomeExchange
Shared Experiences	Leisure Services provided by private entities, mostly locals, e.g., city tours by a resident. In general, commercial platforms connect private and commercial entities	Coolcousins Fromigo
Shared Gastronomy	Local food provided by private entities, mostly locals, but not in a restaurant, e.g., food provided by a private entity in his/her apartment. In general, commercial platforms connect private and commercial entities	Eatwith Foody
Shared Transportation	Transportation services provided or rented by private entities, e.g., a private person using his/her private car or a person renting his/her private boat. In general, commercial platforms connect private and commercial entities	Hiyacar Forestcar
Social Networks	Social networks (generic and tourism-specific solutions) providing content and influencing touristic purchase decisions	Ventoura Mojo Mobility
Software Technology Providers	Companies providing tourism-specific software solutions, e.g., hotel management software	Hotel Runner
Tourism Experience Provider: Accommodation	Commercial providers of hospitality services, e.g., a hotel, pension, campground, etc.	James Villas
Tourism Experience Provider: Activities & Attractions	Providers of touristic activities and attractions in the form of experiences, in general, all POIs	Pure Boats London Duck Tours
Tourism Experience Provider: Gastronomy	Gastronomy provides culinary experiences at visitor attractions and touristic destinations	The Chef’s Cut
Tourism Experience Provider: Transportation (public & private)	Commercial providers of transportation services, e.g., airlines, bus tours, trains. These can be public or private entities	Mister Fly Enjoy Car Hire
Tourist	Tourists consuming a variety of experiences provided. Tourists are travelers on vacation and individuals on business trips	

**Fig. 1.**
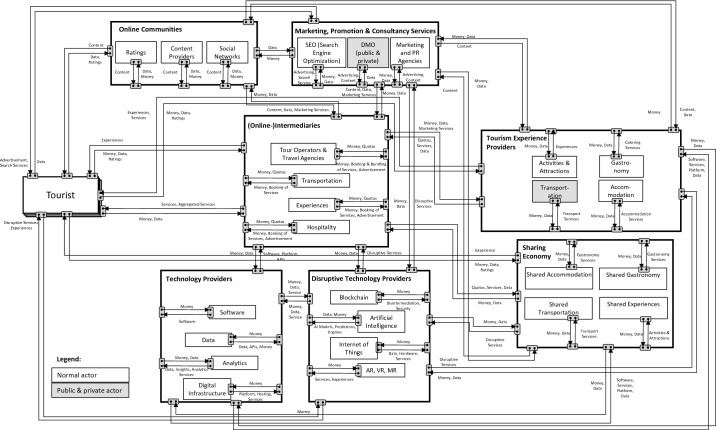
Generic value network of tourism as e3-value model (author’s illustration)

The central roles in the current tourism ecosystem are still tourists, intermediaries, and tourism experience providers. The tourism domain’s generic network shows that the industry transformed into a heterogeneous, multi-sided network: Next to this “core” of the ecosystem, sharing economy services substitute a share of experiences provided previously only by tourism experience providers. This role’s importance has grown within the last decade, challenging existing actors. Specialized marketing, promotion, and consultancy services reflect a purely B2B relationship toward intermediaries and tourism experience providers. Online communities play a special role, capturing tourists’ content and emotions and influencing touristic purchase decisions. Technology providers ensure the ecosystem’s functioning by operating digital infrastructure and offering relevant software solutions and relevant data for the ecosystem. Next to these, disruptive technology providers evolve, using such technologies as AR to provide new experiences for tourists, as well as B2B services such as the Internet of Things (IoT) and blockchain-based solutions. Disruptive technology providers challenge existing actors, mostly the roles of tourism experience providers, as well as technology providers. Figure 1 presents the resulting overview of the ecosystem.

### Innovation Patterns and Strategic Implications of the Tourism Ecosystem

Today’s tourism ecosystem still consists of a core of tourists, intermediaries, and tourism experience providers. Yet, the industry demonstrates high dynamism as well as an increasing number of collaborative business models. Tourists are modern consumers whose expectations are high and influenced by a variety of factors, including further roles, such as online communities. Nowadays, tourists consume “experiences” rather than services, reflecting their increasing expectations. Aggregated bundles of services must complement each other to provide a sound and complete tourist experience [23, 25]. As such, tourists’ purchase decisions are highly influenced by ratings (sometimes even referred to as the “rating economy”) provided by specialized touristic entities (e.g., TripAdvisor), integrated ratings of intermediaries (such as booking.com), but also by content provided in social networks.

Today’s tourists rate almost all their consumed experiences on some platform. At the same time, especially among more tech-savvy tourists, non-tourism related platforms, for example, Instagram, influence decisions such as booking accommodations.

Special in the tourism domain, compared to other domains, is the public sector’s important role. For one thing, public entities are responsible for the governance of heterogeneous actors. Especially for sharing economy services, new rules and mechanisms had to be introduced to balance a thriving local economy and to prevent harm to residents. For example, in Spain, rental of an apartment with Airbnb nowadays requires a special license to improve the management of tourist flow.

At the same time, public entities play a central role not only in governing their local region but also in marketing their destination for touristic providers. Destination Marketing Organizations (DMO) often take this multi-sided role, ensuring governance and, at the same time, promoting their regions and in some cases even offering a booking-platform for small accommodations.

Lastly, if tourists have an unmet need, the market tends to solve this issue by itself, often leading to the creation of new business models and actors. For example, mostly non-tech-savvy individuals operate a large share of European camping sites. Thus, tourists can encounter difficulties finding current information, such as open capacities and data about properties, for instance, “Is the campsite suitable for wheelchairs?” Because in such cases no actor is offering the information, specialized online communities have evolved so that tourists themselves provide and share it.

## Conclusion and Outlook

Dynamic developments in the age of COVID-19 have demonstrated the necessity of having an in-depth understanding of the ecosystem and led to the call for further research [13]. Thus, this paper presents a generic value network of the European tourism ecosystem. Based on a structured content analysis of Crunchbase data of 704 organizations associated with tourism and subsequent validation by expert interviews, we derived 27 distinct roles. Our findings show that the “core” of the ecosystem—tourists, intermediaries, and tourism experience providers—remains the same. Yet nowadays, experiences instead of services are offered, delivering a full package personalized for users’ needs. Next to that core, the role of sharing economy services has increased within the domain. We show the emergence of new, disruptive actors, offering experiences for end-users, as well as pure B2B services. The ecosystem acknowledges the relevance of digital technologies in enabling increased levels of servitization, i.e. in building value for touristic experiences [4], and value co-creation among actors. As such, the availability of data-based services has and still is growing within the tourism ecosystem. In particular analytics services emerge due to more open data in tourism and larger data volumes available [18, 34], offering great benefits for touristic experiences, but increasing the complexity for the players in the industry.

With this research, we contribute to the literature on value networks [24] in the tourism domain. Furthermore, we enhance research on tourism ecosystems with a more computer-science focused, big-picture approach, as proposed by [22]. Prior work has often diminished the use of digital technologies in tourism to e-tourism or smart tourism, positioning this hugely disruptive phenomenon as a sub-discipline of tourism [13]. Yet, a holistic overview of this highly dynamic ecosystem, with different actors and their mutual value interactions was missing as a basis for a more comprehensive understanding of digital technologies’ impacts on tourism as a whole. By identifying ecosystem actors and their interactions, we directly follow the call by [11] to identify roles and value streams in tourism and [13] to enrich tourism research using multidisciplinary approaches. Scholars and practitioners can apply our results to identify opportunities for innovative experiences for tourists, as well as new B2B services. Furthermore, disruptive actors can be uncovered, and existing white spots in the ecosystem can be transparently mapped. Active organizations can apply the model to identify potential threats to their current market positions and evaluate potential opportunities to adapt their business models. Besides that, results may be used as a basis for further analysis of the ecosystem’s ongoing changes induced through technological advancements or external events such as the COVID-19 pandemic.

Naturally, our results have certain limitations and reflect roles and relationships that emerged from our available data, resulting in limited generalizability. Additionally, coding is always partly subjective. To understand interrelations among different actors, we relied on publicly available information, such as reports, press articles, or companies’ websites. However, by validating our results with five experts, the mentioned limitations were mitigated, but not fully eliminated. To generalize our findings further, empirical research is necessary. Besides that, tourism connects various industries. As such, further actors from other ecosystems could be included, especially from the payment and insurance sectors, which also play a vital role in touristic experiences. However, because our goal was to focus deliberately on tourism-actors, these entities are not included in the dataset used and thus not reflected in the resulting ecosystem.

Future research can build on our results and enhance the network with concrete market shares. If actual revenue shares among different actors were understood in more detail, it would be possible to make objective statements about power shares within the industry. Based on this, the dynamics within this ecosystem can be studied, i.e., understanding how these shares will shift in the future and how the business models employed by providers will evolve [29]. Besides existing actors’ importance, studying actual models of services and experience-provision can give valuable insights. One interesting trend observed in tourism, compared to other industries, is the importance of the public sector as well as the integration of local communities’ into service provision. Future research can provide valuable insights into whether and how these entities can be better integrated.

## Footnotes

1 Crunchbase is accessible via www.crunchbase.com. We used a premium account for data collection.
